# Role of saliva use during masturbation in the transmission of *Chlamydia trachomatis* in men who have sex with men

**DOI:** 10.1017/S0950268821001941

**Published:** 2021-09-09

**Authors:** Xianglong Xu, Eric P.F. Chow, David Regan, Jason J. Ong, Richard T. Gray, Pingyu Zhou, Christopher K. Fairley, Lei Zhang

**Affiliations:** 1 China Australia Joint Research Center for Infectious Diseases, School of Public Health, Xi'an Jiaotong University Health Science Centre, Xi'an, Shaanxi, People's Republic of China; 2 Melbourne Sexual Health Centre, Alfred Health, Melbourne, VIC, Australia; 3 Central Clinical School, Faculty of Medicine, Nursing and Health Sciences, Monash University, Melbourne, VIC, Australia; 4 Centre for Epidemiology and Biostatistics, Melbourne School of Population and Global Health, The University of Melbourne, Melbourne, VIC, Australia; 5 The Kirby Institute, University of New South Wales, Sydney, NSW, Australia; 6 STD clinic, Shanghai Dermatology Hospital, Shanghai, China; 7 Department of Epidemiology and Biostatistics, College of Public Health, Zhengzhou University, Zhengzhou, Henan, People's Republic of China

**Keywords:** Anatomical, chlamydia, masturbation, mathematical, modelling, saliva, transmission

## Abstract

Masturbation is a common sexual practice in men, and saliva is often used as a lubricant during masturbation by men who have sex with men. However, the role of saliva use during masturbation in the transmission of chlamydia is still unclear. We developed population-level, susceptible-infected-susceptible compartmental models to explore the role of saliva use during masturbation on the transmission of chlamydia at multiple anatomical sites. In this study, we simulated both solo masturbation and mutual masturbation. Our baseline model did not include masturbation but included transmission routes (anal sex, oral-penile sex, rimming, kissing and sequential sexual practices) we have previously validated (model 1). We added masturbation to model 1 to develop the second model (model 2). We calibrated the model to five clinical datasets separately to assess the effects of masturbation on the prevalence of site-specific infection. The inclusion of masturbation (model 2) significantly worsened the ability of the models to replicate the prevalence of *C. trachomatis*. Using model 2 and the five data sets, we estimated that saliva use during masturbation was responsible for between 3.9% [95% confidence interval (CI) 2.0–6.8] and 6.2% (95% CI 3.8–10.5) of incident chlamydia cases at all sites. Our models suggest that saliva use during masturbation is unlikely to play a major role in chlamydia transmission between men, and even if it does have a role, about one in seven cases of urethral chlamydia might arise from masturbation.

Highlights

- This is the first mathematical modelling study exploring the role of saliva when it is used as a lubricant for solo masturbation and mutual masturbation in the transmission of *Chlamydia trachomatis*.
- Our models suggest that saliva use during masturbation is unlikely to play a major role in chlamydia transmission between men.
- Our models suggest that about 1 in 7 cases of urethral chlamydia might arise from masturbation.

## Introduction

*Chlamydia trachomatis* (*C. trachomatis*) is a common sexually transmitted infection in men who have sex with men (MSM) [1–3] that is considered to be primarily transmitted between men by condomless penile-anal sex [4, 5]. The evidence for other transmission routes and the potential role of saliva is limited [5]. A prospective cohort of community-based HIV negative MSM in Sydney, Australia, reported that incident urethral chlamydia was associated with frequent oral sex with ejaculation [6]. And incident anal chlamydia was also associated with frequent receptive rimming [6]. In contrast, another cross-sectional study among MSM in Peru showed that receptive oral-penile sex was not significantly associated with oropharyngeal chlamydia infection [7]. A further mathematical modelling study explored the transmission of *C. trachomatis* in MSM and found that sexual practices involving the oropharynx or saliva (e.g. oral sex or rimming) improved the calibration of the model [8].

Masturbation is a common sexual practice [9], and saliva is often used as a lubricant during masturbation. In a study conducted among 446 MSM attending a sexual health service in Melbourne, 38% of participants reported they had used saliva as a lubricant for solo masturbation, and 33% of participants reported they had used saliva as a lubricant for mutual masturbation [10]. Another Melbourne-based cross-sectional study reported that 48–61% of MSM practised mutual masturbation using saliva as a lubricant with their most recent regular or casual partners [11]. A study reported that the median bacterial DNA load of *C. trachomatis* in the saliva was 446 copies/ml [interquartile ranges (IQR), 204–1390 copies/ml] and that in the tonsillar fossae was 893 copies/swab (IQR, 390–13 224), and 1204 copies/swab (IQR, 330–16 211) in the posterior oropharynx [12]. In a retrospective study of MSM attending the Melbourne Sexual Health Centre, about 2.2% (95% confidence interval (CI) 1.6–2.5) had oropharyngeal chlamydia, and 3.1% (95% CI 2.6–3.7) had urethral chlamydia [3]. Another cross-sectional study of men who had only received fellatio in the previous 4 months suggested that this exposure was the likely source of urethral *C. trachomatis* in men[13]. Taken together, the results of these studies raise the possibility that sexual practices involving saliva or saliva contamination may carry *C. trachomatis* bacterial from the oropharynx to the urethra and contribute to chlamydia transmission.

However, to date, no study has investigated the role of saliva use during masturbation in the transmission of chlamydia. Research into the role of saliva use during masturbation may be beneficial to understand chlamydia transmission in MSM. Considering the potential role that the oropharynx and saliva play in sex between men, we used mathematical models to test the role of saliva used during solo masturbation and mutual masturbation in transmitting *C. trachomatis* in MSM.

## Methods

### Mathematical model

We developed the population-level, susceptible-infected-susceptible (SIS) compartmental models of *C. trachomatis* transmission based on previous published anatomical site-specific models [8, 14–16]. To reflect *C. trachomatis* infection status, the model incorporated eight states/compartments: (1) susceptible MSM; (2) infection at the oropharynx only; (3) infection at the urethra only; (4) infection at the anorectum only; (5) infection at the oropharynx and urethra only; (6) infection at the oropharynx and anorectum; (7) infection at the urethra and anorectum; and (8) infection at the oropharynx and urethra and anorectum (see Supplementary Fig. S1).

### Site-specific datasets for model calibration

We identified five available studies with single-site infection and multisite infection of *C. trachomatis* using nucleic acid amplification test (NAAT) from four countries: (1) 4888 MSM attending Melbourne Sexual Health Centre in 2018 and 2019 [16]; (2) MSM surveillance data (271 242 consultations) from all STI clinics in the Netherlands in 2008–2017 [17]; (3) a community sample of 1610 MSM in Thailand in 2015–2016 [18]; (4) 393 MSM attending STD & HIV care clinics in the USA in 2016–2017 (Pol BVD) [19]; and (5) 179 MSM with HIV in the USA in 2014–2016 [20] (see Supplementary Table S1).

### Saliva as a lubricant for solo and mutual masturbation

**Fig. 1.**
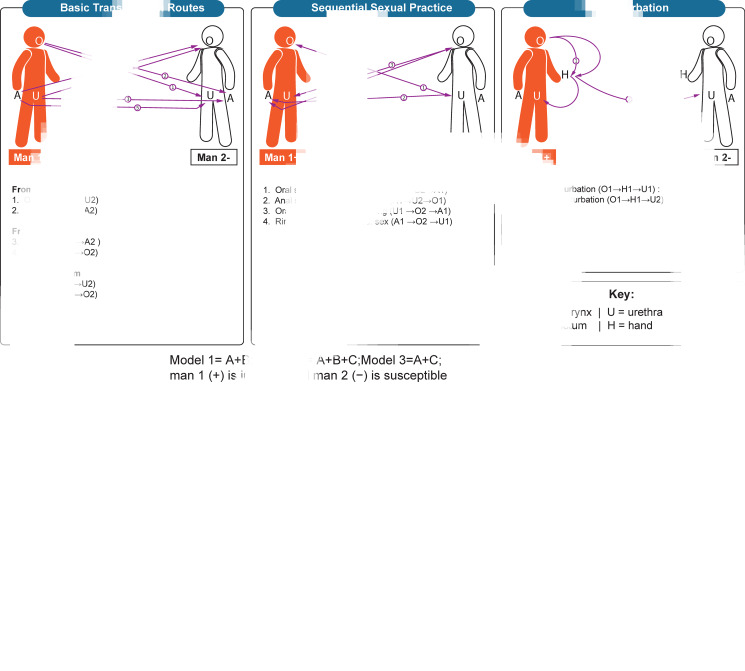
Transmission routes of *Chlamydia trachomatis*. (A) Basic transmission routes. oral sex, anal sex and rimming; (B) Sequential sexual practices including oral sex followed by anal sex (or vice versa) and followed by oral−anal sex (rimming) or vice versa; (C) Masturbation. Saliva uses as a lubricant for solo masturbation, and saliva uses as a lubricant for mutual masturbation.

In this study, we simulated both solo masturbation and mutual masturbation (Fig. 1). Our models assumed that there was limited or no *C. trachomatis* bacteria lost during saliva as a lubricant for solo and mutual masturbation, and a hand with saliva and placed on the penis could transmit *C. trachomatis* to the same extent as from the oropharynx. Transmission from solo masturbation occurs when a man uses his saliva as a lubricant and transmits *C. trachomatis* from his oropharynx to his own urethra and thereby cause multisite infection of the oropharynx and urethra. Transmission from mutual masturbation is when a man uses his saliva as a lubricant on his partner's penis and transmits *C. trachomatis* from his oropharynx to his partner's urethra and thus cause urethra infection of his partner. The hand acts as a mediator during the above two sexual practices and carries saliva with *C. trachomatis* to his own urethra or his partner's urethra.

### Model development

We used our published *C. trachomatis* model as the baseline model (model 1) [8] and established two additional models to test the effect of masturbation on the transmission of *C. trachomatis* (outlined in the text in Fig. 1). Our previous model found that anal sex, penile−oral sex, rimming, sequential oral/anal sex and sequential oral sex/riming could calibrate the single site infection at the oropharynx, urethra and anorectum and multisite infection. Model 1, therefore, included the transmission routes of anal sex, oral sex, rimming, sequential oral/anal sex and sequential oral sex/riming. Model 2 included anal sex, oral sex, rimming, sequential oral/anal sex, sequential oral sex/riming and solo and mutual masturbation. Model 3 included anal sex, oral sex, rimming and solo and mutual masturbation. To further evaluate the effect of masturbation on model calibration, we established model 4 by removing masturbation and removing sequential sexual practices to model 1. Differential equations for these models are provided in the supplementary materials.

### Model parameterisation and fitting

We used previously published sexual practice and *C. trachomatis* infection progression data in the assumption to inform the input parameter values for the models. Masturbation parameters included frequency of solo masturbation, frequency of mutual masturbation, saliva used for solo masturbation and proportion of saliva use for mutual masturbation (Supplementary Table S2).

We used MATLAB R2019a (The Mathworks, Natick, MA) to conduct numerical simulations and perform the statistical analysis. We sampled parameter space from predefined ranges using Latin Hypercube Sampling (Supplementary Table S2). In this way, we generated 1000 parameter sets for model simulation. Using each sampled set of parameters as the initial points, we simulated the transmission model. The models were fitted to clinical diagnosis data of *C. trachomatis* at single-site infection (i.e. oropharynx, urethra and anorectum) and multisite infection (oropharynx and urethra together, oropharynx and anorectum together, urethra and anorectum together, oropharynx and urethra and anorectum together). We calculated the root mean squared error (RMSE) between the simulated prevalence of chlamydia and the clinical data points used to evaluate the goodness of fit for single-site and multisite infections, with a lower value of RMSE indicates a better fit. We fitted to all five site-specific datasets individually. We used the Matlab optimisation function *fmincon* to minimise the RMSE during the simulation process for each of the 1000 parameter sets [21]. Each simulation's output was a set of calibrated prevalence (with minimised RMSE) and the corresponding set of optimised input parameters. Out of these simulations, we sorted the simulation outputs in descending order of the RMSE. The top 10% of simulations with the least RMSE were used to generate the 95% CIs of the model outputs.

We used the calibrated models to estimate *C. trachomatis* incidence. In brief, we estimated the new *C. trachomatis* infections at any given time and calculated the ratio between the number of new infections and the number of susceptible men. We assessed the relative incidence (proportion of incidence cases) based on person-years incidence to explore the relative importance of different anatomical sites (oropharynx, urethra and anorectum) or solo and mutual masturbation for *C. trachomatis* infection by solo and mutual masturbation. Therefore, we calculated this proportion as the rate of incidence cases by solo or mutual masturbation (numerators) and the sum of all *C. trachomatis* cases in a year (denominator). The study methods have been reported previously [8, 14, 16].

### Statistical analysis

We also conducted an independent-sample *t*-test to analyse the difference of RMSE between the two models, where we consider a *P* value <0.05 to indicate a statistically significant difference. Although the *P* value indicates whether there is an effect, it does not reveal the effect's size [22]. With a sufficiently large sample, statistical tests will almost always show a significant difference unless there is no effect. Therefore, we also used Cohen's *d* to estimate the effect size of the RMSE difference between the two models [22, 23]. Even if the RMSE was significantly different, a small Cohen's *d* means that the two distributions' actual overlap is small. Therefore, we used both significant RMSE and large Cohen's *d* to confirm the difference between the two models. Effect sizes were classified as large (Cohen's *d* ⩾ 0.8)[22].

### The sensitivity of the model to masturbation parameters

Due to the variations in the frequency of sexual practices in MSM [14], we conducted sensitivity analyses to assess the impact of varying the selected parameters (frequency of solo masturbation and mutual masturbation and proportion of saliva used for solo masturbation and mutual masturbation) on the uncertainty of model calibration and the incidence. Sensitivity analysis was performed using the Latin hypercube sampling method, and we confirmed the model's robustness concerning small parameter perturbations [24]. (Further details are provided in the supplementary Table S3).

## Results

### Model calibration

**Fig. 2.**
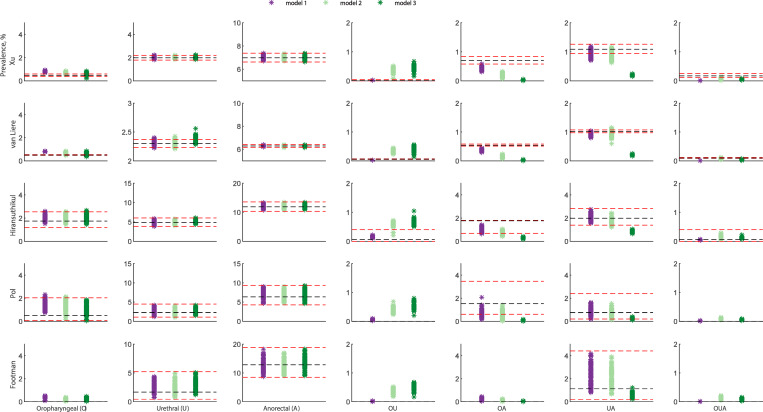
Model calibration and chlamydia data fitting to site-specific infection across five different datasets. Dataset 1: Xu; Dataset 2: van Liere; Dataset 3: Hiransuthikul; Dataset 4: Pol; Dataset 5: Footman. Red dashed lines denote 95% CIs; Black dashed lines denote the mean value; Model 1: Anal sex, oral sex, rimming, sequential oral/anal sex and sequential oral sex/riming; Model 2: Model 1 + masturbation; Model 3: removing sequential practices and adding masturbation.

We built model 2 by adding masturbation to model 1. Model 2 was able to replicate single site infection at the oropharynx, urethra and anorectum and multisite infection across five datasets. However, model 2 overestimated the clinical multisite infection data at the oropharynx and urethra together across five datasets and underestimated the clinical multisite infection data at the oropharynx and anorectum together across three datasets (Fig. 2).

We built model 3 by adding masturbation and removing sequential sexual practices to Model 1. We found that model 3 could replicate the prevalence of chlamydia at single anatomical sites but overestimated the clinical multisite infection data at the oropharynx and urethra together across five datasets and underestimated the clinical multisite infection data at the urethra and anorectum together across three datasets.

### Evaluation of model calibration

**Fig. 3.**
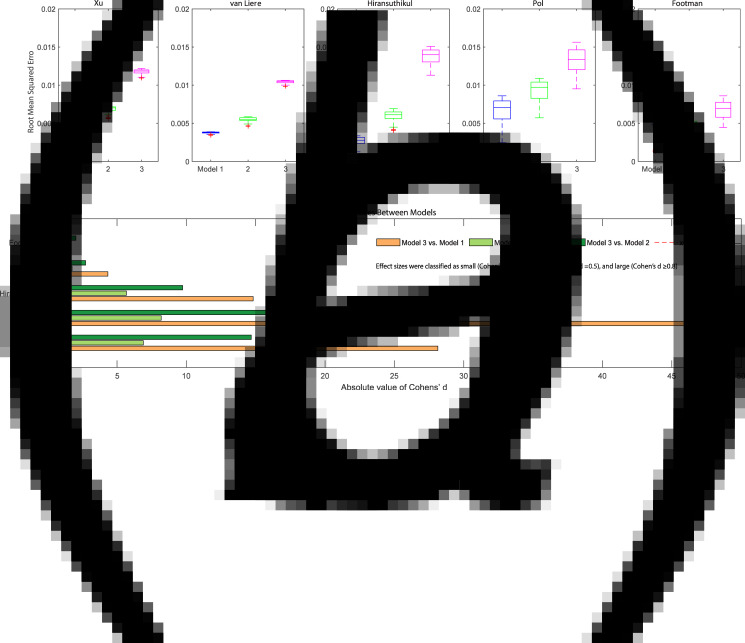
RMSError (A) and effect size (B) of calibrated chlamydia models with or without masturbation across five different datasets. Dataset 1: Xu; Dataset 2: van Liere; Dataset 3: Hiransuthikul; Dataset 4: Pol; Dataset 5: Footman. Model 1: Anal sex, oral sex, rimming, sequential oral/anal sex and sequential oral sex/riming; Model 2: Model 1 + masturbation; Model 3: removing sequential practices and adding masturbation.

For model 2, the inclusion of saliva use during masturbation worsened the goodness-of-fit for the model in terms of matching the clinical infection data. Model 2 had a significantly higher RMSE than model 1 (*P* value <0.01 for all five datasets), and in four of the datasets, the effect size was large (Cohen's *d* > 0.8). Fig. 3.

For model 3, the inclusion of saliva use during masturbation and removal of sequential sexual practices also worsened the goodness-of-fit for the model (model 3 *vs.* model 1). Model 3 had significantly higher RMSE than model 1 (*P* value <0.01 for all five datasets), and the effect size between the two models was large (Cohen's *d* > 0.8 for all five datasets). Similarly, the removal of sequential sexual practices also worsened the goodness-of-fit for the model. Compared with model 2, model 3 demonstrated significantly higher RMSE, and the effect size between the two models was large (Cohen's *d* > 0.8 for all five datasets). (Fig. 3; Supplementary Table S4).

### Estimated incidence of infection from saliva use during solo masturbation and mutual masturbation

**Fig. 4.**
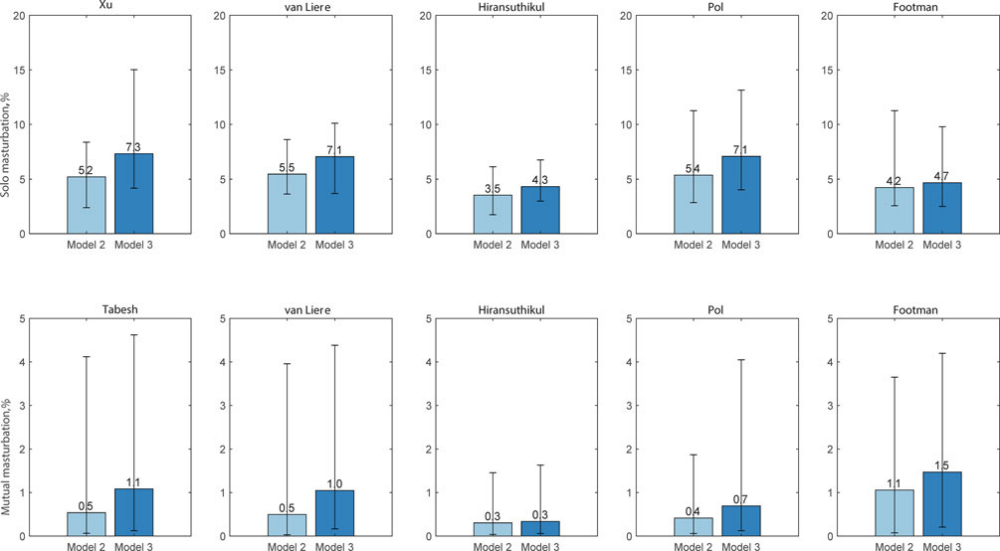
Estimated proportion of chlamydia incidence by masturbation (%). Dataset 1: Xu; Dataset 2: van Liere; Dataset 3: Hiransuthikul; Dataset 4: Pol; Dataset 5: Footman. Model 2: anal sex, oral sex, rimming, sequential oral/anal sex and sequential oral sex/riming + masturbation); Model 3: anal sex, oral sex, rimming and masturbation.

Using model 2, we estimated that the proportion of incident chlamydial cases from the use of saliva during masturbation was responsible for between 3.9% (95% CI 2.0–6.8) and 6.2% (95% CI 3.8–10.5) using the five data sets. Furthermore, saliva use during solo masturbation was responsible for between 3.5% (95% CI 1.7–6.1) and 5.5% (95% CI 3.6–8.6) across five datasets; while saliva use during mutual masturbation was responsible for between 0.3% (95% CI 0.0–1.5) and 0.7% (95% CI 0.1–4.0) across five datasets (Fig. 4; Supplementary Table S5). We also used model 3 to estimate chlamydia incidence from saliva use during solo masturbation and mutual masturbation in five datasets in the supplementary materials. We also estimated the relative incidence of chlamydia at the oropharynx, anorectum or urethra (Supplementary Fig. S2). The three models' estimated proportions of infection incidence at the oropharynx, urethra and anorectum were similar (Supplementary Fig. S2; details in the supplemental materials).

### Sensitivity analysis

Compared with model 4, the inclusion of masturbation (model 3) also worsened the goodness-of-fit for the model. Model 3 had significantly higher RMSE than model 4 (*P* value <0.01 for all five datasets), and the effect size between the two models was large (Cohen's *d* > 0.8 for three datasets). (Supplementary Table S4; details in the supplemental materials).

Varying the frequency of masturbation did not alter our conclusions related to *C. trachomatis* model calibration and incidence estimation. Sensitivity analysis of the RMSE of the calibrated model 2 across five different datasets showed similar results, and Cohen's *d* statistic was less than 0.8 across three datasets. We found Cohen's *d* > 0.8 in two datasets (e.g. increased to double the proportion of MSM using saliva for solo masturbation; decreased to half the days of the frequency of solo masturbation) (Supplementary Fig. S3, Fig. S4).

## Discussion

This is the first mathematical modelling study exploring the role of saliva when it is used as a lubricant for masturbation in the transmission of *C. trachomatis* in men. Our study shows that the inclusion of the transmission route of using saliva as a lubricant for masturbation worsened the ability of the models to replicate the prevalence of *C. trachomatis* at the oropharynx, urethra and anorectum reported in clinical datasets. When we included masturbation, the proportion of incident infections attributable to masturbation was relatively low compared to other sexual practices. Our study suggests masturbation using saliva as a lubricant has a negligible role in chlamydia transmission in men who have sex with men. However, it is important to acknowledge that no empirical data or published studies address this question. Our findings need to be confirmed in epidemiological studies. These studies would initially involve observational epidemiological studies that include masturbation as an exposure. Our study could provide some guidance for future studies on the role of saliva use during masturbation in the transmission of *C. trachomatis*.

Our mathematical models suggest that masturbation involving the saliva may be unnecessary to replicate the prevalence of *C. trachomatis* at multiple anatomical sites in MSM as reported in clinical datasets. This finding is consistent with the study by Cornelisse *et al*., which reported that using saliva as a lubricant for anal sex is not a risk factor for anorectal chlamydia in MSM [25]. Therefore, we conclude that *C. trachomatis* may be less likely to be transmitted via masturbation using saliva. Our findings are consistent with chlamydia's higher affinity for columnar epithelium rather than the squamous epithelium that constitutes most of the oropharynx [26]. A study also indicated that there might be inhibitors in saliva against chlamydia [27]. A previous study suggested that transmission routes other than just oral and anal sex may be necessary to explain the *C. trachomatis* infection at more than one site [8]. Our findings confirm that sequential sexual practices are more important for transmitting *C. trachomatis* than masturbation [8]. This modelling study preliminarily explored the role of masturbation involving saliva in the transmission of *C. trachomatis*. Future empirical studies will be needed to confirm our model findings, including studies assessing the viability of *C. trachomatis* in saliva as well as empirical research to explore the role of sequential sexual practices on the transmission of *C. trachomatis*.

Our results show that saliva use during masturbation plays a negligible role in chlamydia incidence, given that the estimated chlamydia incidence attributed to masturbation was lower than other sexual practices. When adding sequential sexual practices and masturbation in our model, we predicted that about 3.9–6.2% of new cases of chlamydia might arise from masturbation across the five-calibration data set. Using the five data sets, model 2 estimated that 10.2–18.2% of urethral chlamydia infections might arise from masturbation. Furthermore, adding masturbation in the model did not significantly alter the relative proportions of chlamydia incidence at the oropharynx, urethra and anorectum. These results may explain the epidemiological data suggesting why oropharyngeal chlamydia is not common [8, 17, 18, 20, 28].

Our study has some limitations. First, there is very little empirical data about masturbation in men, including its frequency, duration and exactly how saliva is used for solo masturbation or mutual masturbation. This absence of these empirical data will have created considerable uncertainly in our model. The considerable variability in reports about key variables related to masturbation, including even its frequency of masturbation, highlights how personal the issue is and how social desirability bias may impact the studies attempting to measure these behaviours [29]. Also, there may be considerable differences in the reporting of masturbation from individuals from different cultural backgrounds [30]. To address the variability in masturbation practices, we conducted sensitivity analyses for the models concerning the frequency of masturbation and the relative proportions of solo masturbation and mutual masturbation. Second, our findings are limited by the current understanding of the C. trachomatis bacteria in the saliva. We assumed that viable *C. trachomatis* organisms could exist in saliva and could be transmitted via masturbation involving the saliva. However, to the best of our knowledge, no studies have assessed the viability of *C. trachomatis* in saliva, so we do not know if it is even potentially plausible. We hope that this research will encourage more research to explore the role of saliva on the transmission of *C. trachomatis*. Third, we assumed that the members of the MSM population mixed homogeneously in our models. Fourth, we acknowledge that there may be other sex practices we did not include in our models. For example, group sex was not included in our models. Finally, there are few data on multisite infection of *C. trachomatis* available.

## Conclusion

Our models suggest that saliva use during masturbation is unlikely to play a major role in chlamydia transmission between men, and even if it does have a role, about one in seven cases of urethral chlamydia might arise from masturbation. Under this context, we hope our work could encourage further empirical research to explore the role of the oropharynx and saliva on the transmission of chlamydia. Our findings need to be confirmed in epidemiological studies.

## Data Availability

All data analysed during this study are included in this article and its additional file.

## Supplementary material

For supplementary material accompanying this paper visit http://dx.doi.org/10.1017/S0950268821001941. click here to view supplementary material
