# Supplementary material for: Role of saliva use during masturbation in the transmission of Chlamydia trachomatis in men who have sex with men
Source: Epidemiol Infect. 2021 Sep 9;149:e216. doi: 10.1017/S0950268821001941 (PMC8506448; doi:10.1017/S0950268821001941)
Supplement: Supplementary file 1 [file S0950268821001941sup001.docx]

**Supplementary Materials: Role of saliva use during masturbation in the transmission of *Chlamydia trachomatis* in men who have sex with men**

# Literature review

We searched PubMed, up to the 13^th^ of January 2021, for reports of studies assessing the role that masturbation plays in transmitting *C. trachomatis*. We used the search terms (chlamydia [Title/Abstract] OR *C. trachomatis* [Title/Abstract]) AND (masturbation [Title/Abstract] OR masturbation [MeSH]). Of the eight identified sources, no study assessed the role of masturbation in the transmission of *C. trachomatis*.

# Methods

## **Study design**

To reflect the C. trachomatis infection status, the model incorporated eight compartments: (1) susceptible MSM; (2) infection at the oropharynx only; (3) infection at the urethra only; (4) infection at the anorectum only; (5) infection at the oropharynx and urethra only; (6) infection at the oropharynx and anorectum; (7) infection at the urethra and anorectum; and (8) infection at the oropharynx and urethra and anorectum.

**
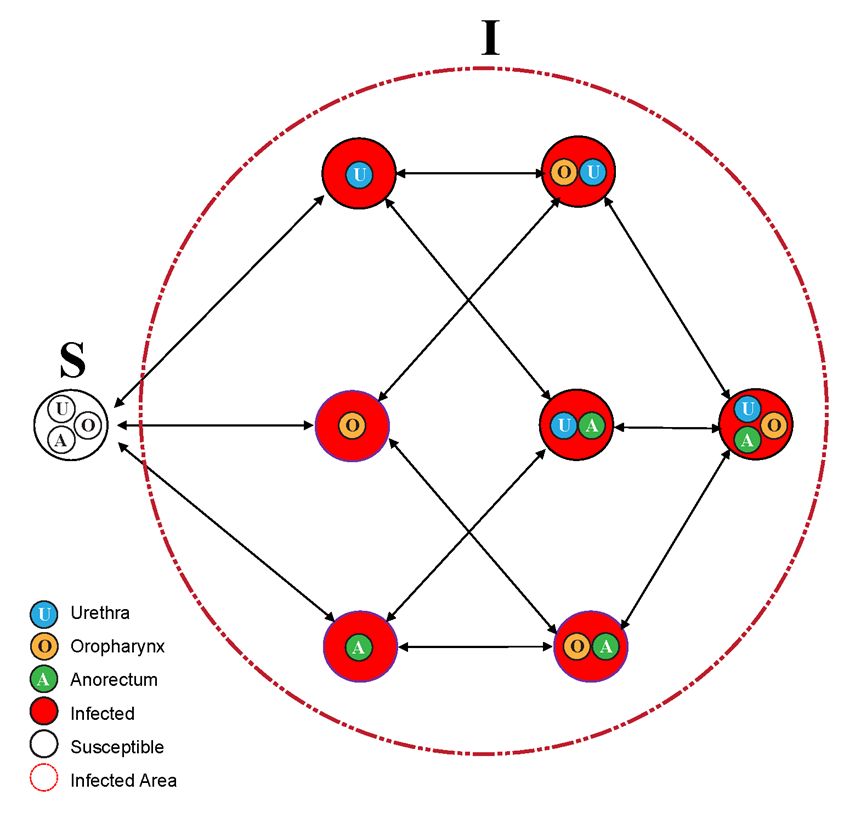
**

### **Supplementary Figure S1**. A compartmental model for the transmission dynamics of *Chlamydia trachomatis* in men who have sex with men.

U: only urethral infections; O: only oropharyngeal infections; A: only anorectal infections; Anorectum (A); OU: only oropharyngeal and urethral infections; UA: only urethral and anorectal infections; OA: only oropharyngeal and anorectal infections; OUA: oropharyngeal, urethral and anorectal infections; arrow signifies the direction of infection and clearance.

**Differential equations**

S = S(t) is the number of susceptible MSM;

I = I(t) is the number of infected MSM;

I_o_ is the number of MSM with oropharyngeal infection only;

I_u_ is the number of MSM with urethral infection only;

I_a_ is the number of MSM with rectal infection only;

I_ou_ is the number of MSM with oropharyngeal and urethral infection only;

I_ua_ is the number of MSM with rectal and urethral infection only;

I_oa_ is the number of MSM with oropharyngeal and rectal infection only;

I_oua_ is the number MSM with oropharyngeal, rectal, and urethral infection;

P_o_ is the *Chlamydia trachomatis* prevalence of infected only at oropharyngeal;

P_u_ is the *Chlamydia trachomatis* prevalence of infected only at urethral;

P_a_ is the *Chlamydia trachomatis* prevalence of infected only at rectal site;

P_ou_ is the *Chlamydia trachomatis* prevalence of infected only at oropharyngeal and urethral sites;

P_ua_ is the *Chlamydia trachomatis* prevalence of infected only at rectal and urethral sites;

P_oa_ is the *Chlamydia trachomatis* prevalence of infected only at oropharyngeal and rectal sites;

P_oua_ is the *Chlamydia trachomatis* prevalence of infected at oropharyngeal, rectal, and urethral sites.

*P* represents the prevalence of *Chlamydia trachomatis;*

$\beta$represents the per-act transmission;

$C$is the percentage of condom use in anal intercourse;

$\varepsilon_{c}$ is the efficacy of condom in preventing transmission of sexually transmitted infections.

$f$ is the frequency of sexual acts that may facilitate transmission. $f$is calculated based on the frequency of sexual acts data [1].

λ: rate of conversion from susceptible to infected individuals, it is a function of per-act transmission probability, frequency of sex acts, condom use and condom efficacy; the product of λ and site-specific prevalence defines the ‘force of infection’ at the specific site;

$\lambda_{ij}$: the rate of conversion from the site i to j;

λ*_ao_*: rimming (anorectum to oropharynx);

λ*_oa_*: rimming (oropharynx to anorectum);

λ*_au_*: anal sex (anorectum to urethra);

λ*_ua_*: anal sex (urethra to anorectum);

λ*_uo_*: oral sex (urethra to oropharynx);

λ*_ou_*: oral sex (oropharynx to urethra);

**The rate of conversion at various sites due to sequential sexual practices**

λ*_ooa_*: conversion at anorectum due to sequential oral sex followed by anal sex;

λ*_aoa_*: conversion at oropharynx due to sequential anal sex followed by oral sex;

λ*_uua_*: conversion at anorectum due to sequential oral sex followed by riming;

λ*_aua_*: conversion at urethra due to sequential riming followed by oral sex;

λ*_ohu1_*: conversion at urethra due to solo masturbation;

λ*_ohu_2*: conversion at urethra due to mutual masturbation;

$\beta_{ij}$: The per-act transmission probability from the site i to j;

$\varepsilon_{c}$: The efficacy of condom in preventing transmission of infection;

C: The percentage of condom use in anal sex;

$f_{ij}$: The frequency of sexual practices from the site i to j (including oral sex, anal sex, kissing, and rimming);

γ: The rate of infection clearance;

γ_u_: The rate of oropharyngeal infection clearance;

γ_a_: The rate of anorectal infection clearance;

γ_o_: The rate of urethral infection clearance

**Force of infection**

The force of infection $\Lambda$takes the following form[1, 2]:

$$\Lambda=\lambda\cdot P$$

$$\lambda=({1-\left( 1-\beta\cdot(1-\varepsilon_{c}\cdot C) \right)}^{\frac{f}{2}})$$

$$N=S+I_{o}+I_{u}+I_{a}+I_{ou}+I_{ua}+I_{oa}+I_{oua}$$

$$P_{o}=I_{o}/N$$

$$P_{u}=I_{u}/N$$

$$P_{a}=I_{a}/N$$

$$P_{ou}=I_{ou}/N$$

$$P_{ua}=I_{ua}/N$$

$$P_{oa}=I_{oa}/N$$

$$P_{oua}=I_{oua}/N$$

$$P_{o\_all}=P_{o}+P_{ou}+P_{oa}+P_{oua}$$

$$P_{a\_all}=P_{a}+P_{oa}+P_{ua}+P_{oua}$$

$$P_{u\_all}=P_{u}+P_{ou}+P_{ua}+P_{oua}$$

$$P_{ou\_all}=P_{ou}+P_{oua}$$

$$P_{ua\_all}=P_{ua}+P_{oua}$$

$$P_{oa\_all}=P_{oa}+P_{oua}$$

$$a1=\lambda_{ao}\boldsymbol{\cdot}P_{a\_all}+\lambda_{uo}{\boldsymbol{\cdot}P}_{u\_all}$$

$$a2=\lambda_{oa}\boldsymbol{\cdot}P_{o\_all}+\lambda_{ua}{\boldsymbol{\cdot}P}_{u\_all}$$

$$a3=\lambda_{ou}{\boldsymbol{\cdot}P}_{o\_all}+\lambda_{au}{\boldsymbol{\cdot}P}_{a\_all}$$

**Differential equations of model 1: with sequential sex practice but without masturbation**

**Compartment S**

$$\frac{dP_{s}}{dt}=-a1\cdot P_{s}+\gamma_{o}\cdot P_{o}-a2\cdot P_{s}+\gamma_{a}\cdot P_{a}-a3\cdot P_{s}+\gamma_{u}\cdot P_{u}$$

**Compartment O: Oropharynx**

$$\frac{dP_{o}}{dt}=a1\cdot P_{s}-\gamma_{o}\cdot P_{o}-a2\cdot P_{o}+\gamma_{a}\cdot P_{oa}-a3\cdot P_{o}+\gamma_{u}\cdot P_{ou}-\lambda_{ooa}\cdot P_{o}$$

**Compartment U: Urethral**

$$\frac{dP_{u}}{dt}=a3\cdot P_{s}-\gamma_{u}\cdot P_{u}-a1\cdot P_{u}+\gamma_{o}\cdot P_{ou}-a2\cdot P_{u}+\gamma_{a}\cdot P_{ua}-\lambda_{uua}\cdot P_{u}$$

**Compartment A: Anorectum**

$$\frac{dP_{a}}{dt}=a2\cdot P_{s}-\gamma_{a}\cdot P_{a}-a1\cdot P_{a}+\gamma_{o}\cdot P_{oa}-a3\cdot P_{a}+\gamma_{u}\cdot P_{ua}-\lambda_{aoa}\cdot P_{a}-\lambda_{aua}\cdot P_{a}$$

**Compartment OU: Oropharynx & Urethra**

$$\frac{dP_{ou}}{dt}=a1\cdot P_{u}-\gamma_{o}\cdot P_{ou}+a3\cdot P_{o}-\gamma_{u}\cdot P_{ou}-a2\cdot P_{ou}+\gamma_{a}\cdot P_{oua}$$

**Compartment OA: Oropharynx & Anorectum**

$$\frac{dP_{oa}}{dt}= a2\cdot P_{o}-\gamma_{a}\cdot P_{oa}+a1\cdot P_{a}-\gamma_{o}\cdot P_{oa}-a3\cdot P_{oa}+\gamma_{u}\cdot P_{oua}+\lambda_{ooa}\cdot P_{o}+\lambda_{aoa}\cdot P_{a}$$

**Compartment UA: Urethra & Anorectum**

$$\frac{dP_{ua}}{dt}=a2\cdot P_{u}-\gamma_{a}\cdot P_{ua}+a3\cdot P_{a}-\gamma_{u}\cdot P_{ua}-a1\cdot P_{ua}+\gamma_{o}\cdot P_{oua}+\lambda_{uua}\cdot P_{u}+\lambda_{aua}\cdot P_{a}$$

**Compartment OUA: Oropharynx & Urethra & Anorectum**

$$\frac{dP_{oua}}{dt}=a2\cdot P_{ou}-\gamma_{a}\cdot P_{oua}+a1\cdot P_{ua}-\gamma_{o}\cdot P_{oua}+a3\cdot P_{oa}-\gamma_{u}\cdot P_{oua}$$

**Differential equations of model 2: model 1 + masturbation**

**Compartment S**

$$\frac{dP_{s}}{dt}=-a1\cdot P_{s}+\gamma_{o}\cdot P_{o}-a2\cdot P_{s}+\gamma_{a}\cdot P_{a}-a3\cdot P_{s}+\gamma_{u}\cdot P_{u}- \lambda.ohu2{\boldsymbol{\cdot}P}_{o\_all}$$

**Compartment O: Oropharynx**

$$\frac{dP_{o}}{dt}=a1\cdot P_{s}-\gamma_{o}\cdot P_{o}-a2\cdot P_{o}+\gamma_{a}\cdot P_{oa}-a3\cdot P_{o}+\gamma_{u}\cdot P_{ou}-\lambda_{ooa}\cdot P_{o}- \lambda.ohu1\cdot P_{o}$$

**Compartment U: Urethral**

$$\frac{dP_{u}}{dt}=a3\cdot P_{s}-\gamma_{u}\cdot P_{u}-a1\cdot P_{u}+\gamma_{o}\cdot P_{ou}-a2\cdot P_{u}+\gamma_{a}\cdot P_{ua}-\lambda_{uua}\cdot P_{u}\boldsymbol{+}\lambda.ohu2{\boldsymbol{\cdot}P}_{o\_all}$$

**Compartment A: Anorectum**

$$\frac{dP_{a}}{dt}=a2\cdot P_{s}-\gamma_{a}\cdot P_{a}-a1\cdot P_{a}+\gamma_{o}\cdot P_{oa}-a3\cdot P_{a}+\gamma_{u}\cdot P_{ua}-\lambda_{aoa}\cdot P_{a}-\lambda_{aua}\cdot P_{a}-\lambda.ohu2{\boldsymbol{\cdot}P}_{o\_all}$$

**Compartment OU: Oropharynx & Urethra**

$$\frac{dP_{ou}}{dt}=a1\cdot P_{u}-\gamma_{o}\cdot P_{ou}+a3\cdot P_{o}-\gamma_{u}\cdot P_{ou}-a2\cdot P_{ou}+\gamma_{a}\cdot P_{oua}\boldsymbol{+(}\lambda.ohu1\cdot P_{o}\boldsymbol{)}$$

**Compartment OA: Oropharynx & Anorectum**

$$\frac{dP_{oa}}{dt}= a2\cdot P_{o}-\gamma_{a}\cdot P_{oa}+a1\cdot P_{a}-\gamma_{o}\cdot P_{oa}-a3\cdot P_{oa}+\gamma_{u}\cdot P_{oua}+\lambda_{ooa}\cdot P_{o}+\lambda_{aoa}\cdot P_{a}\boldsymbol{-}(\lambda.ohu1\cdot P_{oa}\boldsymbol{)-}\lambda.ohu2{\boldsymbol{\cdot}P}_{o\_all}$$

**Compartment UA: Urethra & Anorectum**

$$\frac{dP_{ua}}{dt}=a2\cdot P_{u}-\gamma_{a}\cdot P_{ua}+a3\cdot P_{a}-\gamma_{u}\cdot P_{ua}-a1\cdot P_{ua}+\gamma_{o}\cdot P_{oua}+\lambda_{uua}\cdot P_{u}+\lambda_{aua}\cdot P_{a}\boldsymbol{+}\lambda.ohu2{\boldsymbol{\cdot}P}_{o\_all}$$

**Compartment OUA: Oropharynx & Urethra & Anorectum**

$$\frac{dP_{oua}}{dt}=a2\cdot P_{ou}-\gamma_{a}\cdot P_{oua}+a1\cdot P_{ua}-\gamma_{o}\cdot P_{oua}+a3\cdot P_{oa}-\gamma_{u}\cdot P_{oua}+(\lambda.ohu1\cdot P_{oa})+ \lambda.ohu2{\boldsymbol{\cdot}P}_{o\_all}$$

**Differential equations of model 3: model 2 - sequential sex practices**

**Compartment S**

$$\frac{dP_{s}}{dt}=-a1\cdot P_{s}+\gamma_{o}\cdot P_{o}-a2\cdot P_{s}+\gamma_{a}\cdot P_{a}-a3\cdot P_{s}+\gamma_{u}\cdot P_{u}- \lambda.ohu2{\boldsymbol{\cdot}P}_{o\_all}$$

**Compartment O: Oropharynx**

$$\frac{dP_{o}}{dt}=a1\cdot P_{s}-\gamma_{o}\cdot P_{o}-a2\cdot P_{o}+\gamma_{a}\cdot P_{oa}-a3\cdot P_{o}+\gamma_{u}\cdot P_{ou}- \lambda.ohu1\cdot P_{o}$$

**Compartment U: Urethral**

$$\frac{dP_{u}}{dt}=a3\cdot P_{s}-\gamma_{u}\cdot P_{u}-a1\cdot P_{u}+\gamma_{o}\cdot P_{ou}-a2\cdot P_{u}+\gamma_{a}\cdot P_{ua}\boldsymbol{+}\lambda.ohu2{\boldsymbol{\cdot}P}_{o\_all}$$

**Compartment A: Anorectum**

$$\frac{dP_{a}}{dt}=a2\cdot P_{s}-\gamma_{a}\cdot P_{a}-a1\cdot P_{a}+\gamma_{o}\cdot P_{oa}-a3\cdot P_{a}+\gamma_{u}\cdot P_{ua}-\lambda.ohu2{\boldsymbol{\cdot}P}_{o\_all}$$

**Compartment OU: Oropharynx & Urethra**

$$\frac{dP_{ou}}{dt}=a1\cdot P_{u}-\gamma_{o}\cdot P_{ou}+a3\cdot P_{o}-\gamma_{u}\cdot P_{ou}-a2\cdot P_{ou}\boldsymbol{+(}\lambda.ohu1\cdot P_{o}\boldsymbol{)}$$

**Compartment OA: Oropharynx & Anorectum**

$$\frac{dP_{oa}}{dt}= a2\cdot P_{o}-\gamma_{a}\cdot P_{oa}+a1\cdot P_{a}-\gamma_{o}\cdot P_{oa}-a3\cdot P_{oa}\boldsymbol{-}(\lambda.ohu1\cdot P_{oa}\boldsymbol{)-}\lambda.ohu2{\boldsymbol{\cdot}P}_{o\_all}$$

**Compartment UA: Urethra & Anorectum**

$$\frac{dP_{ua}}{dt}=a2\cdot P_{u}-\gamma_{a}\cdot P_{ua}+a3\cdot P_{a}-\gamma_{u}\cdot P_{ua}-a1\cdot P_{ua}\boldsymbol{+}\lambda.ohu2{\boldsymbol{\cdot}P}_{o\_all}$$

**Compartment OUA: Oropharynx & Urethra & Anorectum**

$$\frac{dP_{oua}}{dt}=a2\cdot P_{ou}-\gamma_{a}\cdot P_{oua}+a1\cdot P_{ua}-\gamma_{o}\cdot P_{oua}+a3\cdot P_{oa}-\gamma_{u}\cdot P_{oua}+(\lambda.ohu1\cdot P_{oa})+ \lambda.ohu2{\boldsymbol{\cdot}P}_{o\_all}$$

## **Data resource**

### **Table S1.** Site-specific prevalence of chlamydia

| Country/region | Sample size | Prevalence/ Mean value (95%CI) | | | | | | |
| --- | --- | --- | --- | --- | --- | --- | --- | --- |
|  |  | Oropharynx only | Urethral only | Rectal  only | Oropharynx and urethra both | Oropharynx and rectum both | Urethra and rectum both | Oropharynx and urethra and rectum both |
| **Xu** [2] | 4888 | 0.76  (0.54-1.06) | 1.86  (1.51-2.29) | 7.53  (6.81-8.31) | *Footnote b,*  0.00  (0.00-0.00) | 1.19  (0.91-1.55) | 1.19  (0.91-1.55) | 0.16  (0.07-0.33) |
| **Pol** [3] | 393 | 0.51  (0.09-2.03) | 2.29  (1.12-4.46) | 6.36  (4.24-9.37) | *Footnote b,*  0.00  (0.-0.00) | 1.53  (0.62-3.47) | 0.76  (0.20-2.40) | *Footnote b,*  0.00  (0.00-0.00) |
| **van Liere** [4] |  | 0.51  (0.48-0.54) | 2.30  (2.23-2.37) | 6.29  (6.17-6.41) | 0.06  (0.05-0.07) | 0.54  (0.51-0.58) | 1.02  (0.97-1.07) | 0.10  (0.09-0.12) |
| **Hiransuthikul** [5] | 1610 | 1.74  (1.18-2.54) | 4.84  (3.87-6.03) | 11.86  (10.34-13.56) | 0.06  (0.00-0.40) | 1.76  (0.69-1.80) | 1.99  (1.39-2.83) | 0.06  (0.00-0.40) |
| **Footman** [6] | 179 | *Footnote* b,  0.00  (0.-0.00) | 1.68  (0.44-5.22) | 12.85  (8.49-18.86) | *Footnote b,*  0.00  (0.-0.00) | *Footnote b,*  0.00  (0.-0.00) | 1.12  (0.19-4.41) | *Footnote b,*  0.00  (0.-0.00) |

**Footnote:**

1. MSHC started screening for throat chlamydia in mid-2017, so we used chlamydia data for 2018-19.
2. Since the positivity is zero, we assumed the upper bound of 95% confidence interval was 1/1000000 (one person in a million) and the lower bound of 95% was 0.0000000 in the simulation.

## **Model parameters**

### **Table S2.** Biological and behavioural data of *Chlamydia trachomatis* for model parameterization and calibration

| **Parameters** | **Value (uncertainty bounds)** | **References/Remarks** |
| --- | --- | --- |
| %, Consistent condom usage in anal sex in past 12 months | 46.90 (34.50- 59.30) | [1] |
| %, Condom efficacy in preventing transmission | 87.50 (80.00-95.00) | [1, 7] |
| Frequency of oral sex (days) | 13.53 (0.00-28.11) | [1] |
| Frequency of rimming (days) | 38.57 (0.00-80.15) | [1] |
| Frequency of anal sex (days) | 26.44 (0.00-54.94) | [1] |
| weeks, Infection duration of *Chlamydia trachomatis* at the throat (asymptomatic infection) | 95.29 (24.87-245.44) | [8, 9]. |
| weeks, Infection duration of *Chlamydia trachomatis* at urethral (symptomatic infection) | 1.57 (1.00-2.00) | Footnote *a*, [10] |
| weeks, Infection duration of *Chlamydia trachomatis* at urethral (asymptomatic infection) | 57.72 (19.60-158.08) | [11] |
| %, proportion of urethral infections that are asymptomatic | 85.00 (75.00-95.00) | Footnote *b*, [12] |
| %, Proportion of anal *Chlamydia trachomatis* infections that are asymptomatic | 87.50 (60.41-97.80) | Footnote *c*, [13] |
| weeks, Infection duration of *Chlamydia trachomatis* at the anus | 82.68 (50.02-134.16) | [11] |
| %, Proportion of MSM received throat swab in the past 12 months | 79.65 (63.70-95.60) | Footnote *d*, [14] |
| %, Proportion of MSM received anal swab in the past 12 months | 79.65 (63.70-95.60) | Footnote *d*, [14] |
| %, Proportion of MSM received urine test in the past 12 months | 79.65 (63.70-95.60) | Footnote *d*, [14] |
| %, proportion of 'oral sex and anal sex' in the same sex episode | 29.41 (24.82-34.00) | Footnote *e*, [15] |
| %, proportion of 'oral sex and rimming' in the same sex episode | 70.5 (67.94-72.94) | Footnote *f,* [16] |
| %, proportion of receptive oral sex (individual’s mouth in contact with a partner’s penis) followed by partner's insertive anal sex (individual’ penis in contact with partner’ anus) | 80.00 (80.00-80.00) | Footnote *g* [17] |
| %, proportion of insertive oral sex (individual’s penis in contact with a partner’s mouth) followed by partner's insertive rimming (individual’s mouth in contact with a partner’s anus) | 80.00 (80.00-80.00) | **Estimate from Melbourne Sexual Health Centre** |
| **Masturbation parameters** |  |  |
| Frequency of solo masturbation (days) | 2.0 (1.4-3.5) | Footnote h, [18] |
| Frequency of mutual masturbation (days) | 5.36 (3.5 – 80.15) | Footnote i, [19, 20]. |
| Proportion of saliva use for solo masturbation, % | 37.7 (33.3-42.3) | Footnote j, [21]. |
| Proportion of saliva use for mutual masturbation, % | 33.6 (29.4-38.1) | Footnote k, [21]. |

**Footnote**:

1. The duration time is estimated by the time between the first symptom and treatment. Among Men, the mean time was 11 Days =1.57 weeks, range (7-14) days.
2. We estimated the value from a previous study. A case-control study of MSM attending Seattle & King County STD Clinic between 2001 and 2013 found that clinicians diagnosed a total of 6464 urethral infections, only 61% of chlamydial infections were symptomatic and asymptomatic chlamydial infections were 39%.
3. 16 MSM was infected with anal *Chlamydia trachomatis*, of which 14 were asymptomatic.
4. We used the proportion of gay and bisexual men attending sexual health clinic for a chlamydia test in 2017 (95.60%) as the upper bound. We used the proportion of gay and bisexual men attending general practice clinic tested for chlamydia in 2017 (63.70%) as the lower bound. The mean value of proportion = (upper bound+ lower bound)/2.
5. The proportion of 'oral sex and anal sex' in the same sex episode was not available from published data. We used the prevalence of insertive anal sex (34.00%) as the upper bound. We also used the value of the prevalence of anal sex (34.00%) multiply the prevalence of oral sex (73.00%) as the lower bound. The mean value of proportion = (upper bound+ lower bound)/2.
6. The proportion of 'oral sex and rimming' in the same sex episode was not available from published data. About 70.5% (95%CI67.94-72.94) reported rimming. According to the advice of sexually transmitted infections specialist at the Melbourne Sexual Health Centre, rimming is always accompanied by oral sex. We are using the mean value and 95%CI for rimming as the mean and bound for the proportion of 'oral sex and rimming' in the same sex episode.
7. The proportion of receptive oral sex (individual’ mouth in contact with partner’ penis) followed by partner's insertive anal sex (individual’ penis in contact with partner’ anus) was not available from published data. The sequence of receptive oral sex followed by partner's insertive anal sex was most common among oral sex and anal sex combinations. Based on the advice of sexually transmitted infections specialist at the Melbourne Sexual Health Centre, we made the assumption.
8. Males masturbated approximately two to five times a week. We estimated the frequency of solo masturbation was 3.5(2.0-5.0) times per week. Therefore, the estimated frequency of solo masturbation was 2.0 (1.4-3.5) days.
9. According to results of the Durex Global Sex Survey 2005, the frequency of sex in Australia was 108[19]. The proportion of for mutual masturbation was 63.0%[20]. The mean proportion of frequency of mutual masturbation was 365/ (108*63.0%) =365/68=5.36 days. Solo masturbation is more common than mutual masturbation. The lower bound was the upper bound of solo masturbation. The upper bound of (kissing, oral sex, rimming, or anal sex) was 80.15; therefore, we choose this as the upper bound of mutual masturbation.
10. Among 446 participants, the proportion of using saliva as lube when masturbating was 168 (37.7%).
11. Among 446 participants, the proportion of masturbated my partner off using my saliva as lube was 149 (33.4%). The proportion of my partner masturbating me off with his saliva as lube was 151 (33.9%). Therefore, we got the proportion of saliva use for mutual masturbation was (149+151)/446=150 (33.6%).

## **Sensitivity Analysis**

### **Table S3. Masturbation parameters of sensitivity analysis**

| **Group** | **Parameters** | **Values** |
| --- | --- | --- |
| 1 | Increased to double the frequency of solo masturbation | 4.00 (2.80-7.00) |
| 2 | Increased to double the frequency of mutual masturbation | 10.72 (7.00-160.30) |
| 3 | Increased to double the proportion of saliva used for solo masturbation | 75.40 (66.60-84.60) |
| 4 | Increased to double the proportion of saliva use for mutual masturbation | 67.20 (58.80-76.20) |
| 5 | Decreased to half the frequency of solo masturbation | 1.00 (0.70-1.75) |
| 6 | Decreased to half the frequency of mutual masturbation | 2.68 (1.75-40.08) |
| 7 | Decreased to half the proportion of saliva used for solo masturbation | 18.85 (16.65-21.15) |
| 8 | Decreased to half the proportion of saliva use for mutual masturbation | 16.8 (14.7-19.05) |

# Results

### **Table S4.** Root mean squared error and Cohen's d effect size of calibrated chlamydia models with or without masturbation across five different datasets

| Models | Root mean squared error | T test, Absolute value of Cohens’ d |
| --- | --- | --- |
| **Xu** [2] |  |  |
| Model 1 | 0.0048(95%CI 0.0042 to 0.0050) | Ref. |
| Model 2 | 0.0070(95%CI0.0058 to 0.0073) | Model 2 vs. model 1, p value <0.01, d = 6.90 |
| Model 3 | 0.0118(95%CI0.0111 to 0.0122) | Model 3 vs. model 1, p value <0.01, d= 28.13  Model 3 vs. model 2, p value<0.01, d= 14.68 |
| Model 4 | 0.0113(95%CI 0.0106 to 0.0116) | Model 4 vs. model 1, p value<0.01, d = 27.20  Model 4 vs. model 3, p value<0.01, d = 2.03 |
| **van Liere** [4] |  |  |
| Model 1 | 0.0038(95%CI0.0035 to 0.0039) | Ref. |
| Model 2 | 0.0055(95%CI 0.0047 to 0.0058) | Model 2 vs. model 1, p value<0.01, d = 8.19 |
| Model 3 | 0.0105(95%CI 0.0100 to 0.0106) | Model 3 vs. model 1, p value<0.01, d = 48.02  Model 3 vs. model 2, p value<0.01, d = 21.55 |
| Model 4 | 0.0101(95%CI 0.0098 to 0.0102) | Model 4 vs. model 1, p value<0.01, d = 61.25  Model 4 vs. model 3, p value <0.01, d = 2.45 |
| **Hiransuthikul** [5] |  |  |
| Model 1 | 0.0028(95%CI 0.0016 to 0.0033) | Ref. |
| Model 2 | 0.0061(95%CI0.0045 to 0.0069) | Model 2 vs. model 1, p value<0.01, d = 5.68 |
| Model 3 | 0.0140(95%CI 0.0119 to 0.0151) | Model 3 vs. model 1, p value<0.01, d = 14.82  Model 3 vs. model 2, p value<0.01, d= 9.73 |
| Model 4 | 0.0131(95%CI 0.0090 to 0.0140) | Model 4 vs. model 1, p value<0.01, d = 10.10  Model 4 vs. model 3, p value<0.01, d = 1.11 |
| **Pol** [3] |  |  |
| Model 1 | 0.0070(95%CI0.0028 to 0.0086) | Ref. |
| Model 2 | 0.0097(95%CI0.0062 to 0.0109) | Model 2 vs. model 1, p value<0.01, d = 1.76 |
| Model 3 | 0.0134(95%CI0.0098 to 0.0155) | Model 3 vs. model 1, p value<0.01, d= 4.34  Model 3 vs. model 2, p value<0.01, d= 2.74 |
| Model 4 | 0.0125(95%CI 0.0097 to 0.0142) | Model 4 vs. model 1, p value <0.01, d = 3.97  Model 4 vs. model 3, p value <0.01, d = 0.63 |
| **Footman** [6] |  |  |
| Model 1 | 0.0054(95%CI0.0015 to 0.0058) | Ref. |
| Model 2 | 0.0051(95%CI0.0039 to 0.0053) | Model 2 vs. model 1, p value<0.01, d = 0.48 |
| Model 3 | 0.0070(95%CI0.0046 to 0.0085) | Model 3 vs. model 1, p value<0.01, d = 1.54  Model 3 vs. model 2, p value<0.01, d = 2.03 |
| Model 4 | 0.0076(95%CI 0.0053 to 0.0090) | Model 4 vs. model 1, p value<0.01, d = 2.25  Model 4 vs. model 3, p value <0.01, d = 0.63 |

Note: Chlamydia Model 1: Anal sex, oral sex, rimming, sequential oral/anal sex and sequential oral sex/riming; model 2 (Model 1 + masturbation); (Model 3 removed sequential practices but added masturbation). Dataset 1: Xu; Dataset 2: van Liere; Dataset 3: Hiransuthikul; Dataset 4: Pol; Dataset 5: Footman.

### **Table S5.** Proportion of chlamydia incidence by masturbation across five different datasets

|  | **Proportion of incidence by** **masturbation, %** | | |
| --- | --- | --- | --- |
|  | Solo Masturbation | Mutual masturbation | Overall masturbation |
| **Xu** [2] |  |  |  |
| Model 2 | 5.2 (95%CI 2.4 to 8.4) | 0.5(95%CI 0.1 to 4.1) | 6.0(95%CI 3.0to 10.6) |
| Model 3 | 7.3(95%CI 4.2 to15.0) | 1.1(95%CI 0.1 to 4.6) | 8.8(95%CI 4.4to 17.6) |
| **van Liere** [4] |  |  |  |
| Model 2 | 5.5(95%CI 3.6 to 8.6) | 0.5(95%CI 0.0 to 4.0) | 6.2(95%CI 3.8to 10.5) |
| Model 3 | 7.1(95%CI 3.7 to 10.1) | 1.0(95%CI 0.2 to 4.4) | 8.2(95%CI 4.9to 12.6) |
| **Hiransuthikul** [5] |  |  |  |
| Model 2 | 3.5(95%CI 1.7 to 6.1) | 0.3(95%CI 0.0 to 1.5) | 3.9(95%CI 2.0to 6.8) |
| Model 3 | 4.3(95%CI 3.0 to 6.8) | 0.3(95%CI 0.1 to 1.6) | 4.8(95%CI 3.3to 7.5) |
| **Pol** [3] |  |  |  |
| Model 2 | 5.4(95%CI 2.8 to 11.3) | 0.4(95%CI 0.1 to 1.9) | 5.8(95%CI 3.2to 12.8) |
| Model 3 | 7.1(95%CI 4.0 to 13.1) | 0.7(95%CI 0.1 to 4.0) | 7.8(95%CI 4.3to 15.6) |
| **Footman** [6] |  |  |  |
| Model 2 | 4.2(95%CI 2.6 to 11.3) | 1.1(95%CI 0.1 to 3.6) | 5.7(95%CI 3.1to 13.7) |
| Model 3 | 4.7(95%CI 2.5 to 9.8) | 1.5(95%CI 0.2 to 4.2) | 6.2(95%CI 3.1to 13.2) |

Note: Chlamydia Model 1: Anal sex, oral sex, rimming, sequential oral/anal sex and sequential oral sex/riming; model 2 (Model 1 + masturbation); (Model 3 removed sequential practices but added masturbation). Dataset 1: Xu; Dataset 2: van Liere; Dataset 3: Hiransuthikul; Dataset 4: Pol; Dataset 5: Footman.

### **Table S6.** The model-estimated per-act transmissibility of masturbation

| Models | Solo Masturbation | Mutual masturbation |
| --- | --- | --- |
| **Xu** [2] |  |  |
| Model 2 | 0.0316 (95%CI 0.0145 to 0.0808) | 0.0007(95%CI 0.0001 to 0.0061) |
| Model 3 | 0.0539 (95%CI 0.0261 to 0.2981) | 0.0015(95%CI 0.0002 to 0.0090) |
| **van Liere** [4] |  |  |
| Model 2 | 0.0265 (95%CI 0.0170 to 0.0668) | 0.0006(95%CI 0.0000 to 0.0049) |
| Model 3 | 0.0398(95%CI 0.0185 to 0.0841) | 0.0012(95%CI 0.0002 to 0.0050) |
| **Hiransuthikul** [5] |  |  |
| Model 2 | 0.0109 (95%CI 0.0055 to 0.0278) | 0.0002 (95%CI 0.0000 to 0.0013) |
| Model 3 | 0.0153 (95%CI 0.0081 to 0.0321) | 0.0003(95%CI 0.0000 to 0.0018) |
| **Pol** [3] |  |  |
| Model 2 | 0.0176 (95%CI 0.0058 to 0.0909) | 0.0003 (95%CI 0.0000 to 0.0029) |
| Model 3 | 0.0338 (95%CI 0.0127 to 0.4625) | 0.0007 (95%CI 0.0001 to 0.0066) |
| **Footman** [6] |  |  |
| Model 2 | 0.2269 (95%CI 0.0376 to 0.9952) | 0.0045 (95%CI 0.0003 to 0.0290) |
| Model 3 | 0.2783 (95%CI 0.0621 to 0.9997) | 0.0058 (95%CI 0.0008 to 0.0207) |

Footnote: Dataset 1: Xu; Dataset 2: van Liere; Dataset 3: Hiransuthikul; Dataset 4: Pol; Dataset 5: Footman.


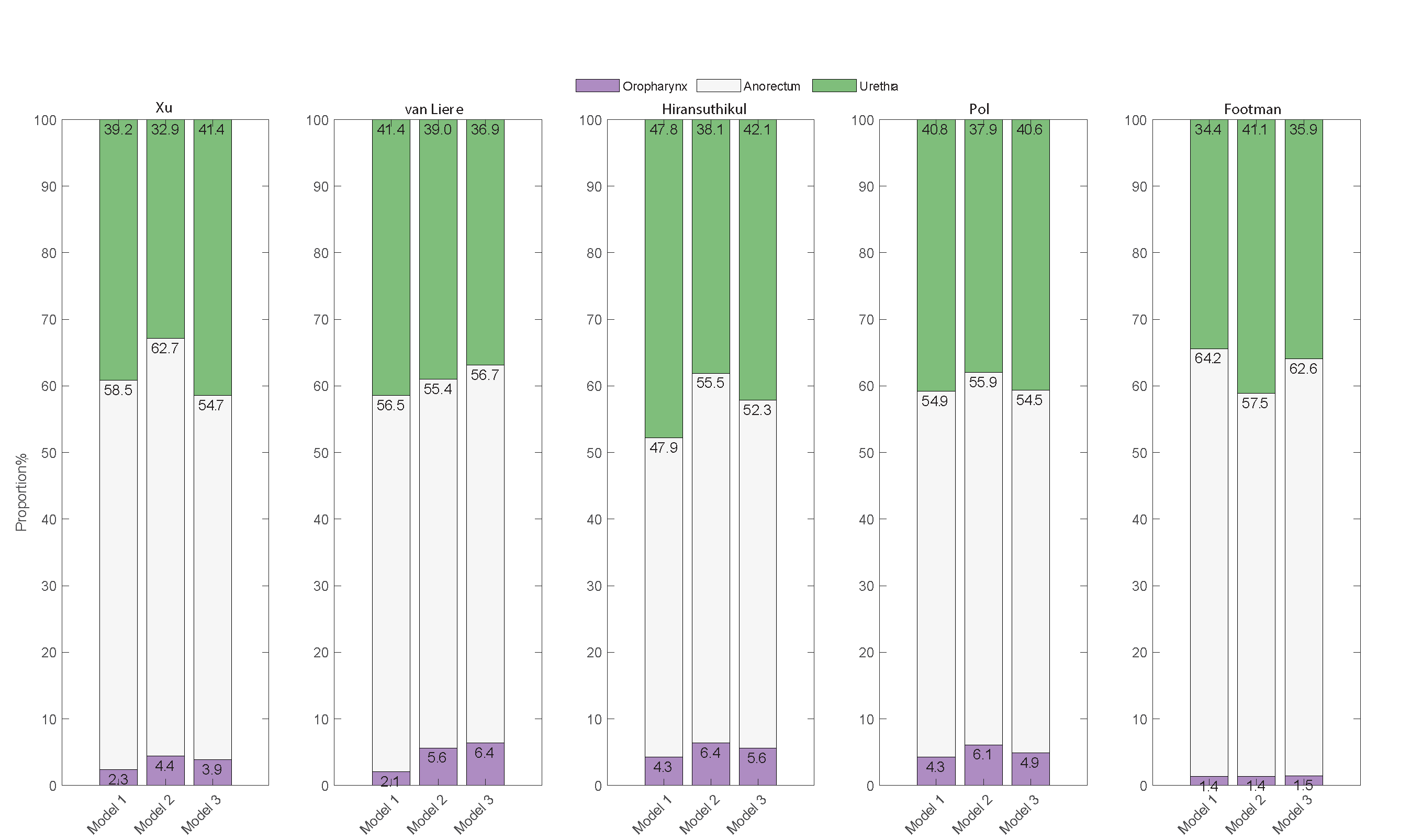


### **Figure S2**. Estimated proportion of chlamydia incidence at oropharynx, urethra and anorectum

Model 1: anal sex, oral sex, rimming, sequential oral/anal sex and sequential oral sex/riming; model 2 (Model 1 + masturbation); (Model 3 removed sequential practices and added masturbation). Dataset 1: Xu; Dataset 2: van Liere; Dataset 3: Hiransuthikul; Dataset 4: Pol; Dataset 5: Footman.

# Sensitivity analysis


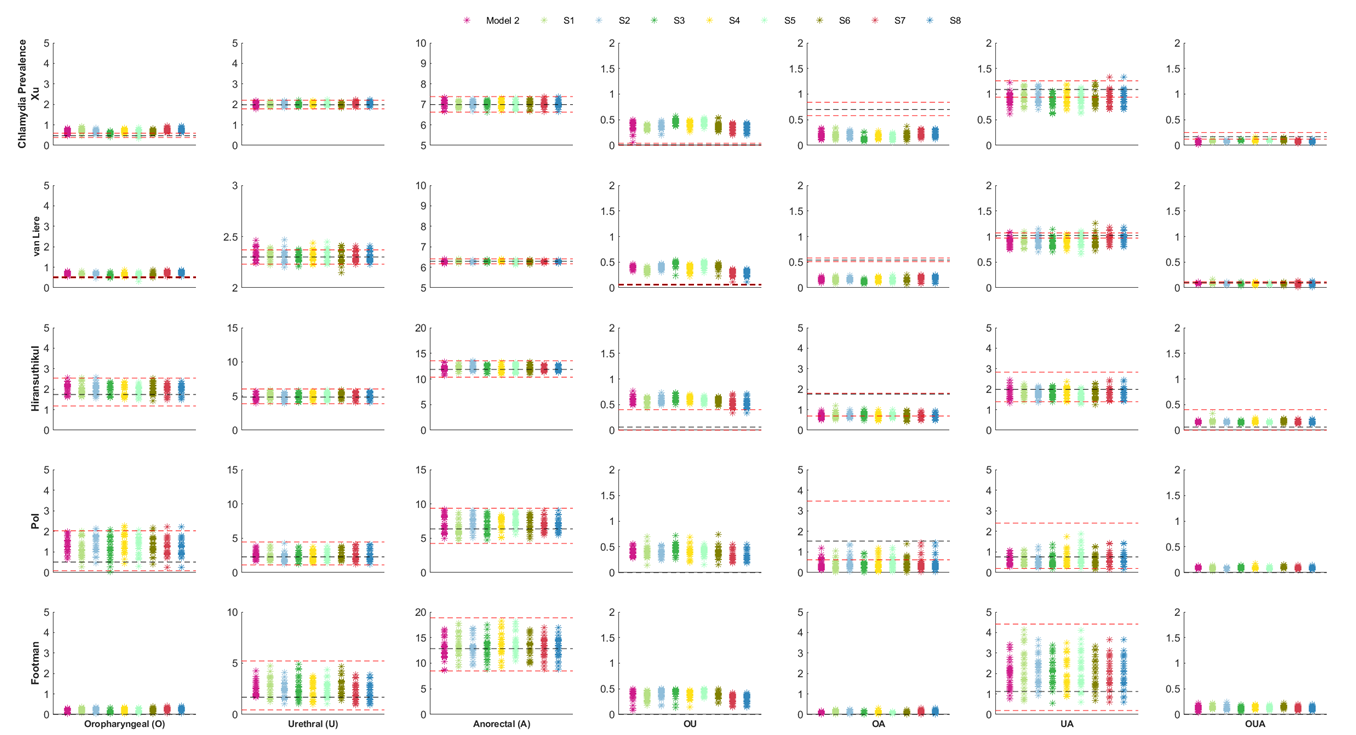


### **Figure S3.** Model 2 calibration and chlamydia data fitting to site-specific infection across five different datasets.

Red dashed lines denote 95% confidence intervals. Black dashed lines denote the mean value; Xu[2], van Liere [4], Hiransuthikul [5], Pol [3], and Footman [6]. 1) S1: increased to double the days of the frequency of solo masturbation; 2) S2: increased to double the days of the frequency of mutual masturbation; 3)S3: increased to double the proportion of saliva used for solo masturbation; 4) S4: increased to double the proportion of saliva use for mutual masturbation; 5) S5: decreased to half the days of the frequency of solo masturbation; 6) S6: decreased to half the days of the frequency of mutual masturbation; 7) S7: decreased to half the proportion of saliva used for solo masturbation; 8) S8: decreased to half the proportion of saliva use for mutual masturbation.


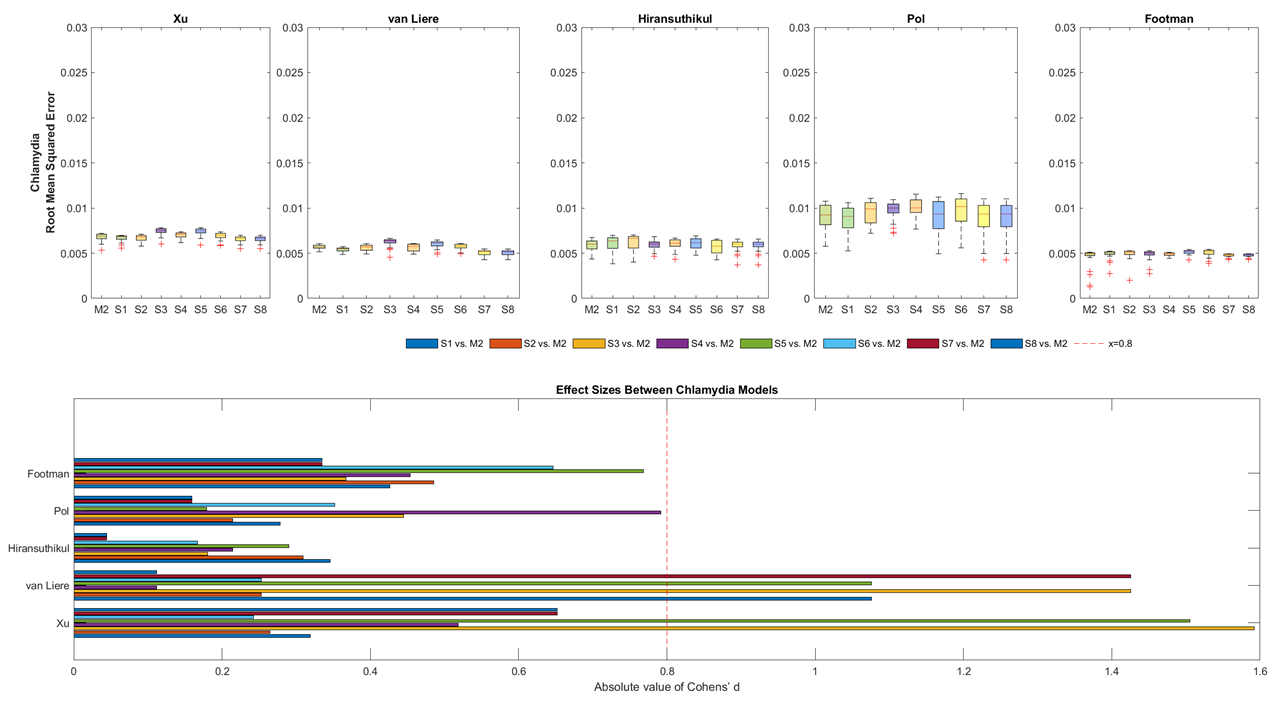


### **Figure S4.** Sensitivity analysis of root mean squared error and effect size of calibrated chlamydia model 2 with or without masturbation across five different datasets

Xu[2], van Liere [4], Hiransuthikul [5], Pol [3], and Footman [6]; 1) S1: increased to double the days of the frequency of solo masturbation; 2) S2: increased to double the days of the frequency of mutual masturbation; 3) S3: increased to double the proportion of saliva used for solo masturbation; 4) S4: increased to double the proportion of saliva use for mutual masturbation; 5) S5: decreased to half the days of the frequency of solo masturbation; 6) S6: decreased to half the days of the frequency of mutual masturbation; 7) S7: decreased to half the proportion of saliva used for solo masturbation; 8) S8: decreased to half the proportion of saliva use for mutual masturbation.

# Reference

(1) **Zhang L, et al.** Neisseria gonorrhoeae Transmission Among Men Who Have Sex With Men: An Anatomical Site-Specific Mathematical Model Evaluating the Potential Preventive Impact of Mouthwash. *Sex Transm Dis* 2017; **44**(10): 586-592.

(2) **Xu X, et al.** Chlamydia trachomatis transmission between the oropharynx, urethra and anorectum in men who have sex with men: a mathematical model. *BMC Med* 2020; **18**(1): 326.

(3) Pol BVD. Extragenital CT/GC in MSM. In. The 20th International Union against Sexually Transmitted Infections- Asia Pacific Conference (IUSTI-AP), 2019.

(4) **van Liere G, et al.** What is the optimal testing strategy for oropharyngeal Neisseria gonorrhoeae in men who have sex with men? Comparing selective testing versus routine universal testing from Dutch STI clinic data (2008-2017). *Clin Infect Dis* 2019.

(5) **Hiransuthikul A, et al.** Correlations of chlamydia and gonorrhoea among pharyngeal, rectal and urethral sites among Thai men who have sex with men: multicentre community-led test and treat cohort in Thailand. *BMJ Open* 2019; **9**(6): e028162.

(6) **Footman A, et al.** Performance of 4 Molecular Assays for Detection of Chlamydia and Gonorrhea in a Sample of Human Immunodeficiency Virus-Positive Men Who Have Sex With Men. *Sex Transm Dis* 2020; **47**(3): 158-161.

(7) **Tuli K, Kerndt PR.** Preventing sexually transmitted infections among incarcerated men who have sex with men: a cost-effectiveness analysis. *Sex Transm Dis* 2009; **36**(2 Suppl): S41-48.

(8) **Templeton DJ, et al.** Prevalence, incidence and risk factors for pharyngeal chlamydia in the community based Health in Men (HIM) cohort of homosexual men in Sydney, Australia. *Sex Transm Infect* 2008; **84**(5): 361-363.

(9) **Chow EP, et al.** Duration of gonorrhoea and chlamydia infection at the pharynx and rectum among men who have sex with men: a systematic review. *Sex Health* 2016; **13**(3): 199-204.

(10) **Andersen B, et al.** Prediction of costs, effectiveness, and disease control of a population-based program using home sampling for diagnosis of urogenital Chlamydia trachomatis Infections. *Sex Transm Dis* 2006; **33**(7): 407-415.

(11) **Jin F, et al.** Incidence and risk factors for urethral and anal gonorrhoea and chlamydia in a cohort of HIV-negative homosexual men: the Health in Men Study. *Sex Transm Infect* 2007; **83**(2): 113-119.

(12) **Barbee LA, et al.** An estimate of the proportion of symptomatic gonococcal, chlamydial and non-gonococcal non-chlamydial urethritis attributable to oral sex among men who have sex with men: a case-control study. *Sex Transm Infect* 2016; **92**(2): 155-160.

(13) **Rebe K, et al.** A Cross Sectional Analysis of Gonococcal and Chlamydial Infections among Men-Who-Have-Sex-with-Men in Cape Town, South Africa. *PLoS One* 2015; **10**(9): e0138315.

(14) Institute. TK. HIV, viral hepatitis and sexually transmissible infections in Australia: annual surveillance report 2018. In, 2018.

(15) **Fairley CK, et al.** Models of gonorrhoea transmission from the mouth and saliva. *The Lancet Infectious Diseases* 2019.

(16) **Chow EPF, et al.** Saliva use as a lubricant for anal sex is a risk factor for rectal gonorrhoea among men who have sex with men, a new public health message: a cross-sectional survey. *Sex Transm Infect* 2016; **92**(7): 532-536.

(17) **Rosenberger JG, et al.** Sexual behaviors and situational characteristics of most recent male-partnered sexual event among gay and bisexually identified men in the United States. *J Sex Med* 2011; **8**(11): 3040-3050.

(18) **Bőthe B, et al.** Hypersexuality, Gender, and Sexual Orientation: A Large-Scale Psychometric Survey Study. *Arch Sex Behav* 2018; **47**(8): 2265-2276.

(19) **Network D**. The Face of Global Sex 2005; 2005.

(20) **Rosenberger JG, et al.** Sexual Behaviors and Situational Characteristics of Most Recent Male‐Partnered Sexual Event among Gay and Bisexually Identified Men in the United States. *The Journal of Sexual Medicine* 2011; **8**(11): 3040-3050.

(21) **Chow EPF, et al.** Antiseptic mouthwash for gonorrhoea prevention (OMEGA): a randomised, double-blind, parallel-group, multicentre trial. *Lancet Infect Dis* 2021.
